# Cystic fibrosis care today—a narrative review based on views of experts, patients and the public

**DOI:** 10.1007/s00508-026-02739-3

**Published:** 2026-04-08

**Authors:** Florian Singer, Anja Maria Schaffer, Michaela Auer, Jakob Auer, Jasmin Pansy, Vanessa Stadlbauer, Ernst Eber

**Affiliations:** 1 Division of Paediatric Pulmonology and Allergology, Department of Paediatrics and Adolescent Medicine, Medical University of Graz, Graz, Austria; 2 Department of Respiratory Medicine, University Children’s Hospital Zurich, University of Zurich, Zurich, Switzerland; 3 Public Patient Involvement Group, Medical University of Graz, Graz, Austria; 4 Kinderärzte Graz Leonhard, Graz, Austria; 5 Division of Gastroenterology and Hepatology, Department of Internal Medicine, Medical University of Graz, Graz, Austria

**Keywords:** Patient and public involvement, Cystic fibrosis, Hygiene, Treatment Burdon, Muco-obstructive Lung Disease

## Abstract

Cystic fibrosis (CF) is one of the most common rare, genetic diseases. Newly available, highly effective modulator therapy (HEMT) improves many domains of CF pathophysiology. Thereby, HEMT challenges current standards of care and prediction models of disease outcomes.

We established a public patient involvement (PPI) group including experts and caregivers of children with CF and collated a narrative review on CF management. This review is based on the views of healthcare professionals and caregivers and summarizes important features of the disease, prevention and treatment which are currently considered relevant for caregivers and Austrian healthcare settings.

## Background

Cystic fibrosis (CF) is one of the most common, rare congenital disorders in Austria. In 2012, the first new targeted medication, known as highly effective modulator therapy (HEMT), was approved. These therapeutics improve the function of many, but not all, affected organ systems. As a result, new aspects have emerged in the understanding and treatment of CF.

Patient and public involvement (PPI) could provide important perspectives on the experiences of people living with or impacted by a chronic health condition. In this review article, experts and family members describe CF disease and the care of people with CF at home, in healthcare and childcare facilities in Austria. The aim of the review article was to collate current evidence and their interpretation on both contextual and personal levels, provided by experts and family members in the format of a narrative review. We describe contemporary evidence and expert and patient perspectives on CF management in an Austrian CF center and subjectively examine the relevance of evidence and current recommendations.

Cystic fibrosis is considered a birth defect, an inborne disability or congenital anomaly in international legislation [1]. It is a monogenic multiorgan disease caused by disease-causing variants in the *cystic fibrosis transmembrane conductance regulator* (*CFTR*) gene [2]. It is the most common life-limiting genetic disease among people of European descent. Worldwide, over 100,000 individuals are affected. In Austria, 877 people with CF are currently registered in the European CF Registry, including 358 children under the age of 18 years [3]. Conventional treatment aims at downstream effects of *CFTR* dysfunction [4]. Without treatment, the majority of children would die within the first years of life; with extensive conventional treatment the average life expectancy is approximately 52 years [5]. Computer simulations suggest a significant increase in life expectancy due to the newly available HEMT [6], which is approved only for specific variants in the *CFTR* gene and age groups, which may vary between national authorities [7]. The most common life-limiting organ manifestations of CF affect the airways, the lungs and the gastrointestinal tract [2].

### Personal viewpoint of a family with two children with CF

The diagnosis of our first son born in 2020 brought the following insights: the increasingly malfunctioning organs of our little wonder will be life-limiting one day. To know this caused feelings of guilt, helplessness and sadness.

The diagnosis of our second child born in 2022 brought additional insights: we can help our children to accept CF as a part of themselves. We work on that attitude thereby experiencing many ups and downs which will always accompany us. Modulator therapy and the research progress bring hope for us. In the following article we provide insights in our development as a family with children with CF.

### Personal viewpoint of a healthcare professional and mother of a daughter with CF

After the first shock caused by the diagnosis following abnormal newborn screening, a time of being torn between my role as a mother and healthcare professional, i.e. consultant for internal medicine and gastroenterology and hepatology, commenced. Because of my research expertise, I was able to search, read and understand the scientific literature; however, it was hard for me to accept the “hard facts” as a mother. Yet, communicating with other parents of children with CF helped me to accept the diagnosis and to implement medical expert knowledge into practice.

## Methods

For this narrative review, we established a public patient involvement (PPI, consumer involvement) group [8]. Experts and families in the CF centre Graz were invited to write a review for the public.

Members of the PPI group were recruited during outpatient visits and through professional networks. Patients, family members, experts, and pediatricians were invited to contribute to a PPI group and a narrative review. Out of five people from the public approached four were willing to engage in the PPI activities. The current PPI group consists of a physician and mother of a school-aged girl with CF, a private practice pediatrician and a couple who are parents of two preschool-aged boys with CF. The research question of this work was on contemporary evidence and personal views on current CF management by involving experts in the field and our newly established PPI group [9]. Literature published from 2000 to 2024 in English and German on CF care was searched in PubMed and Google. Original papers, reviews and consensus recommendations were considered and summarized across all levels of expertise involved in this work. Search terms were: cystic fibrosis, cystic fibrosis lung disease, lung function in cystic fibrosis, cystic fibrosis liver disease (CFLD), cystic fibrosis related diabetes (CFRD), CFTR modulator therapy and cystic fibrosis care. This review involves the PPI group members as authors. Interviews were not performed. In the context of this review, the “involvement” component of PPI refers to activities and research carried out with members of the public. Following informal meetings and written correspondence, the focus of this research was jointly identified, the body of this work was edited and enriched by perspectives on relevant subjects in CF care. Members of the PPI were equal partners in this research and considered for all key decisions during this project [10]. The PPI group provided information on personal experiences, for example, on practicability of hygiene safety measures, and were consulted on all aspects including, for example, wording such as “survival”. The PPI group lead all aspects of personal viewpoints.

### Personal viewpoint of a healthcare professional and mother of a daughter with CF

The *Public and Patient Involvement *initiative is very welcome as it acknowledges affected people, patients and caregivers as experts of the disease. It also acknowledges the importance of patients’ and caregivers’ input across all phases of research, planning and analyses of studies, interpretation of research findings from the caregivers’ perspective and implementation into pragmatic, daily life practice.

### Personal viewpoint of a family with two children with CF

Our comments are not considered medical recommendations. Instead, our statements are meant to provide insight into everyday life of families of children with CF who balance, for example, hygiene suggestions and their children’s individual needs. Every family will develop their own view on hygiene suggestions usually formed by advice from local healthcare experts and the families’ individual weighting and “risk assessment”. Challenges during implementation may arise and depend on distinct health conditions, the families’ mindset, personal living conditions, supportive living environment and socioeconomic background.

## Results

### Respiratory tract

The onset and severity of the respiratory tract disease is poorly associated with *CFTR* variants. Therefore, prediction of respiratory disease outcomes based on genetic information alone is not reliable [11]. Most infants and toddlers develop respiratory symptoms, such as cough and wheezing in the first years of life [12]. Shortly after birth, abnormal structural changes, such as bronchiectasis and functional changes in the airways and lungs can be detected in 60–80% of children with CF using lung imaging techniques (computed tomography, magnetic resonance imaging) and pulmonary function tests (multiple-breath washout) [13, 14]. These changes may progress during the preschool and school years [15]. Children with CF who are not eligible for HEMT experience annual decline in lung function, i.e. −1.5% to −2.2% (percent predicted in forced expiratory volume in 1 s, FEV_1_) per year on average [16]. Bronchiectasis involves airway remodelling and seems only reversible during early stages under HEMT [17]. Respiratory infections caused by pathogens transmitted from human to human and from the environment are key factors in airway remodelling. Chronic airway mucus plugging in both upper and lower airways promotes chronic infection. Important pathogens in CF lung disease include, for example, *Pseudomonas aeruginosa*, non-tuberculous mycobacteria (NTM) and *Aspergillus fumigatus*. Hygiene measures are considered important to minimize the risk of respiratory tract infection in CF [18]. Inhalation therapies and chest physiotherapy have positive effects on the downstream effects of respiratory infections [18].

The upper airways may be affected by mucus obstruction, infection and inflammation thereby causing chronic sinusitis and polyps. Symptoms include blocked nose breathing, sleep disordered breathing, pain and hearing difficulties [19]. Topical mucolytics, steroids and, depending on the upper airway disease severity, surgery may be indicated [18]. The implementation of HEMT can significantly improve mucus clearance and is associated with shifts towards a more typical respiratory microbiome in many patients, although chronic infection can persist [20, 21].

### Personal viewpoint of a family with two children with CF

We are constantly weighing our children’s freedom to play and develop normally vs. (social) limitations due to safety considerations. No visitors with symptoms suggesting a respiratory tract infection? Going shopping with or without a face mask? Which hobbies or everyday activities should be avoided? Should we allow our construction passionate players to dig in soil on the ground? Inhalation and chest physiotherapy are parts of our daily routine. Our children are training their lungs. Dad is training his endurance by running. Everybody exercises because it helps “being strong”. If symptoms occur, if troublesome cough causes nausea or even vomiting, we try to stay calm. In cases of doubt, we contact the CF center. This allows us to gain clarity regarding the next steps. Novel treatment options such as modulators can be motivating, but they also come with new challenges. Hospitalizations and specialized care in both CF and rehabilitation centers, across several weeks can contribute to successful treatment and motivate us too.

### Gastrointestinal tract

#### Intestines

The onset and severity of the intestinal disease is weakly associated with *CFTR* variants. Individual prognosis cannot be reliably predicted from genotype alone. During pregnancy or within the first days of life some children with CF suffer from intestinal obstruction: Meconium plugging or ileus [3]. Depending on the severity of obstruction, ileus is treated either nonsurgically or surgically sometimes involving resection. Possible long-term complications include short bowel syndrome requiring enteral or parenteral nutrition [22] via a central venous line, mild obstipation and severe ileus due to fibrotic adhesions [23]. In some people with CF, intestinal obstruction may occur later in life.

The distal intestinal obstruction syndrome (DIOS) is an incomplete or complete, potentially life-threatening ileus caused by an accumulation of feces at the end of the small intestine. Mild to moderate DIOS forms can be treated conservatively. The quality of life can be reduced as a result of gastroesophageal reflux, abdominal pain, meteorism and social isolation [18]. Using HEMT can reduce intestinal inflammation and relieve gastrointestinal symptoms [24]; prevention of gastrointestinal complications is currently under study.

### Personal viewpoint of a family with two children with CF

Two children with two different “gut stories”: one of our children already experienced an intrauterine intestinal obstruction and required surgery shortly after birth. This resulted in a long hospital stay within the neonatal period and, after all, discharge from hospital with a central venous catheter for parenteral nutrition. Now we know: parenteral nutrition via a central venous catheter is indeed feasible at home; however, everyday life is even more demanding: sterile working like healthcare professionals in the intensive care unit as well as precise scheduling and personnel planning. Professional support, home nursing care, family-support service, close contact with the department of pediatrics and adolescent medicine and the pediatric surgery intensive care unit, specialized pharmacies, medical supply stores, pediatricians, contributed substantially to this success. Working family members voluntarily provide support. Everyone can, no one must.

#### Pancreas

The risk of pancreatic insufficiency is strongly associated with *CFTR* variants, allowing some degree of prognostic estimation, particularly for minimal function variants [25]. In most individuals with CF, irreversible remodelling of the pancreas occurs during pregnancy due to obstruction and scarring. As a result, approximately 90% of individuals with CF develop pancreatic insufficiency within a few weeks of birth, characterized by failure of the digestive enzyme system (exocrine function) [3]. This necessitates lifelong supplementation of digestive enzymes. In the second or third decade of life, the hormonal (endocrine) pancreatic function may also decline [26]. Approximately 26% of individuals with CF in Austria develop insulin-dependent CF related diabetes (CFRD) [3]. Modulator treatment generally does not restore pancreatic function in most individuals; early initiation may preserve or partially improve function in people with selected genotypes but robust evidence is still limited [27].

### Personal viewpoint of a family with two children with CF

Pancreatic enzyme replacement therapy (PERT) improves weight gain but, how to breastfeed a “sleepy drinker” baby with sharp-edged pellets in the mouth? Apparent banality, which required a lot of patience for our first child, besides the completely “normal” (challenging) initial time, during which baby and (exhausted) parents as a family find together. Today, PERT granulate, filled in small sample vessels, accompanies us everywhere: in the handbag, in the car and in various jackets of the children. The second-born is still practicing to swallow capsules. The older one is already presenting his achievements with great pride.

#### Liver

The manifestation of chronic CF liver disease (CFLD) is moderately associated with *CFTR* variants; thus, the genotype provides only partial prognostic information [28, 29]. The CF-related liver disease without cirrhosis occurs on average in 25% of individuals with CF. In the second or third decade of life, 5% of individuals with CF develop cirrhosis [3] and, in addition, 1% receive a liver transplantation as reported in the *European Cystic Fibrosis Society Patient Registry* [3]. Therapeutic options are limited to bile acid modulation (ursodeoxycholic acid), supplementation of fat-soluble vitamins and micronutrients, treatment of secondary complications (portal hypertension, esophageal varices, hepatopulmonary syndrome) and liver transplantation. Liver involvement in CF may improve with HEMT [30, 31]. Currently, established CFLD is not considered reversible by HEMT [32].

#### Mental health

Depression occurs in 8–29% of children with CF and, very rarely, may be exacerbated during HEMT [33, 34]. Depression may affect approximately 37% of mothers and 31% of fathers of people with CF. Increased anxiety occurs in 22% of adolescents with CF as well as in 48% of mothers and 36% of fathers. Pain such as headaches, abdominal pain and chest pain occurs in around 70% of children and adults with CF, associated with a reduction in quality of life [35, 36]. In general, complications of chronic diseases include social isolation and financial risks.

Other CF-related complications include infertility and joint and bone disease [37, 38]. Treatment-related complications include hearing loss and cataracts [39, 40].

### Personal viewpoint of a family with two children with CF

What to prioritize in which social setting, raising CF awareness and education vs. unworried playing? What are caregivers supposed to know about the environment? How do we handle hygiene measures constraining social life, for example, bathing in the pond, play gatherings in the season of infections? How do we encourage our children, thereby avoiding “whitewashing” the situation? “You can join—let us get a face mask!”

Every family, every caregiver answers those questions individually and differently. The answers for those questions can vary, depending on the physical and mental states. Exchange with other parents of children with CF and discussions with physicians, dietitians and therapists offer insights and ideas and broaden perspectives which are of great value for us. We learned to bravely demand respecting CF and its peculiarities, such as insisting that nobody is allowed to drink from the mug of my child, from our social environment. We have developed strategies to protect emotional well-being of our own and our children from CF-related reservations regarding social interactions. These strategies provide safety in managing CF for us as parents and offer orientation for our children. Strong negative emotions of the children, which can occur in the context of a (CF-related) “no”, are allowed and accompanied lovingly but also consequently. Depending on our own condition, this can be more or less challenging.

### Prevention and therapy

#### Hygiene

Hygiene is important in the daily lives of people with CF as well as for their caregivers and families [41–45]. Evidence-based CF-related hygiene recommendations have been published by international expert groups [41–45]; however, current recommendations largely reflect expert consensus, were collated prior to widely available HEMT and can be associated with increased daily effort and social constraints [46–49].

Problematic pathogens are present in natural reservoirs of urban and rural environments. *Pseudomonas aeruginosa* infection is an established risk factor for lung disease progression [50]. Due to activities in the vicinity of these reservoirs that may promote aerosol formation and thereby increase the risk of airborne or smear-contact infection, the following hygiene suggestions apply at home and in childcare settings. The main rationale of suggestions listed below is to reduce exposure to waterborne pathogens such as *Pseudomonas* species.

Humidifiers or plants should not be used, particularly no flower vases or hydroponic plants in the close environment of people with CF. Only dry sand in sandboxes should be used for play. When playing with soil or wet sand, gloves should be worn and hands should be washed afterwards. The use of squirt toys (e.g., rubber ducks, water guns, etc.) should be avoided. A face mask should be worn when engaging in activities generating airborne dust. This includes activities in animal barns and exposure to hay or other dusty environments [41, 45]. Keeping pets and visits of closed spaces harboring animals (e.g., zoos, animal barns, terrarium) are discouraged.

Bathing in swimming ponds or in small bathing ponds with standing water should be avoided [41, 45]. If not recommended against by local authorities, public swimming pools in Austria are considered safe regarding exposure to problematic pathogens.

The following specific hygiene measures should be considered:

- After visible or potential contamination, hands should be washed regularly.
- To avoid aerosol formation while washing hands, the water jet should not directly flow into the opening of the washbasin’s sink.
- To avoid aerosol formation in the bathroom, toilets should be flushed when the toilet lid is closed.
- Soap dispensers should be cleaned before refill.
- Single use paper tissues should be used for drying hands.
- Textiles such as towels, flannels and cloth napkins should only be used when dry and washed regularly at 60 °C.
- Close contact to other people with CF, for example, in childcare centers, should be avoided.
- Organic waste or compost should be stored in dustbins with the lid closed and placed outside the living space at home and in childcare centers and emptied regularly.

During respiratory pathogen endemics or pandemics, people with CF are considered at risk [51]. The use of public transport without protective measures should be avoided [52]; however, as wearing FFP2 masks or rigorous hand hygiene are not feasible in young children, private transport may be one of the few viable options.

Failure to comply with recommended hygiene measures can cause respiratory infections and pulmonary exacerbations (CF-related pneumonia), which may require several weeks of intravenous and inhaled antibiotic treatment. In the long term, non-compliance with hygiene measures could promote chronic respiratory infection with, for example, *Pseudomonas aeruginosa* or *Aspergillus* species, which may cause respiratory symptoms, loss of lung function, progression of lung disease, reduced physical fitness and quality of life, and hospitalizations [53–56]; however, these recommendations are largely based on expert consensus rather than controlled trials.

### Personal viewpoint of a family with two children with CF

Hygiene measures!? They are relevant, constructive, demanding and time-consuming at the same time and can lead to insecurity in certain social environments. Adhering to hygiene recommendations may be particularly challenging for children with CF during participation in group activities in kindergartens, schools, or during visits at their friends’ houses.

Where do I set the line between “play and fun” and “the risk is too high in my view” (soap bubbles, private pools or animal barn visits…)?

Hygiene routines (e.g. rinsing the siphons in the morning) indeed establish in everyday life and take a lot of room in raising and educating our children. Older children and adolescents can already reliably put the hygiene measures into practice. We are looking forward to it! At the same time, as our children grow older, the desire of exploring things on their own and making self-determined decisions regarding which hygiene measures to follow develop. It remains to be seen how we, as parents, can support our boys discovering personal freedom and how serious concerns may become when hygiene measures are not followed.

While hygiene measures are clear-cut, their implementation in daily life remains challenging.

#### Acute therapy

Individuals with CF, like those with asthma, may suffer from hyperresponsive airways. Cold air, exercise, dust, odors, respiratory infections, and other triggers can induce discomfort, coughing, or acute shortness of breath. Especially in those with a co-diagnosis of hyperresponsive airways or asthma, fast-acting bronchodilators (e.g., salbutamol) using a spacer (holding chamber) should be on hand [57, 58]. Occasionally, children with CF who are suffering from a respiratory tract infection may need to take oral antibiotics two to three times daily. In rare cases, severe hemoptysis or pneumothorax, a lung collapse, may occur, which may require surgical intervention [59, 60].

#### Maintenance therapy

Mucolytic and, if indicated, antibiotic inhalation therapy, as well as chest physiotherapy two to three times daily, are essential and are recommended across all ages [18, 47, 61]. The time required for the treatment of chronic respiratory disease, including the preparation and postprocessing of medical devices as well as their application, takes approximately 1–3 hours per day. Some people with mild CF lung disease taking HEMT probably may safely reduce mucolytic treatment [62].

#### Nutrition

The use of PERT provides digestive enzymes and should be used for every fat-containing meal in those suffering from pancreatic insufficiency [63]. In the long term, inadequate PERT may lead to impaired growth and failure to thrive [64]. Skilled PERT applies to childcare settings, which should promote independence and awareness of children. Toddlers are unable to dose and swallow PERT on their own and therefore, they depend on trained individuals. Failure to take PERT may lead to abdominal pain, bloating, diarrhea, anal prolapse and potentially to DIOS. In addition to PERT, individuals with CF should (i) drink enough fluids, (ii) salt their food and (iii) consume high-calorie foods and (iv) take fat-soluble vitamin supplements. Children with pancreatic insufficiency should be fed high-calorie diets to prevent failure to thrive, which is associated with impaired lung growth. Occasionally, additional micronutrients or stool softening laxatives are needed. Modulator treatment often improves weight gain and nutritional status in eligible individuals, yet PERT remains to be continued in most people with CF. The effectiveness of HEMT for the prevention of DIOS remains unclear [65]. Children with CF taking HEMT should ensure adequate fluid and salt intake at all times, yet high-calorie diets are often no longer indicated [66]. With increasing life expectancy and the emergence of obesity, and high calorie and salt intake, individual dietary counselling should be emphasized [66]. The spectrum of fluid and salt deficiency ranges from discomfort due to electrolyte and pH imbalances to life-threatening Pseudo-Bartter syndrome.

### Personal viewpoint of a family with two children with CF

In the first year of life, we often gave in the urge to feed our son the calculated and prepared meal to provide the previously determined amount of energy, thereby promoting adequate body growth and development. Regular exchange with dietitians is always helping us to focus on the needs of our sons and to avoid planning meals only based on highest fat content and highest PERT dosage. In any case, some oil or whipped cream is always on the plate. We use “astronaut food” for the older one as snack on “exciting” days.

Our second son takes advantage of his elucidated and more experienced parents. Exchange and training relieve pressure from us and provide flexibility. In the first year of life, our second son was partly nourished using parenteral (intravenous) preparations. In the beginning, we were not allowed to exceed recommended amounts of oral food, as this may have led to obstruction of the small intestine. Additional operations of the intestine may be necessary.

#### Childcare

For the care of children with CF, there is a need for training and additional workload due to the necessary hygiene and treatment measures [67] as summarized below.

#### Hygiene in childcare facilities

Compliance with current CF-related hygiene measures within the institution should be ensured. People responsible should control hygiene in wet rooms and take measures to ensure that, for example, cooking areas (organic waste) and play behavior (water splash fights, construction sites involving soil, wet sand or clay play) do not put children with CF at risk of respiratory infections. Additional supervision and coordination may be necessary for excursions, as children with CF may be allowed to only partly participate in activities, for example, during visits to animal stables, zoos, biotopes and ponds. Compared to healthy peers, children with CF cannot always engage in the same activities. This requires empathic compensation of constrained activities and social restrictions faced by children with CF; however, these recommendations are largely based on expert consensus rather than controlled trials.

#### Potential therapeutic situations in childcare centers

- Evaluation of the food regarding fat content and observation of the child during eating are necessary. The PERT should be dosed timely and according to the amount of consumed food and fat content and administered before and sometimes in addition during or after the meal.
- Food may need extra salting. High-calorie supplements and vitamin preparations may be necessary.
- Depending on current treatment plans, medication should be taken two to three times per day, i.e. including lunchtime: (1) inhaled bronchodilators using a spacer (holding chamber), (2) inhaled hypertonic saline solution and/or dornase alfa using a nebulizer and/or (3) antibiotics administered orally or by inhalation.

#### Potential emergency situations in childcare centers

The following should be recognized, reported and treated quickly:

- Respiratory symptoms such as coughing up bloody respiratory secretions (hemoptysis) [60, 68] and breathlessness [69, 70].
- General symptoms such as reduced general condition, severe abdominal pain, vomiting and other signs of severe forms of ileus [23], electrolyte imbalance [71], acute pancreatitis [72], or renal stones [73].

### Personal viewpoint of a family with two children with CF

The scope of educational counselling for teachers, educators and kindergarten workers regarding medication plans and hygiene measures varies, depending on the self-reliance of the child. Information folders available at our local CF center provide references. Due to the complexity of the disease of our second son, we had to rely on babysitters and daycare for our 2.5-year-old son since the birth of the younger one. To find a fitting healthcare person was challenging. For support in occasional medical tasks for our second son, such as intravenous infusions of parenteral nutrition and bandage change, associations like the *Hilfswerk* (public social aid) or the *Mobile Kinderkrankenpflege* (mobile childcare) were essential.

And, by the way, our children are developing into wonderful individuals who chose us to accompany them through life.

#### Telehealth

Telehealth has gained increasing attention during the severe acute respiratory syndrome coronavirus 2 (SARS-CoV-2) pandemic and beyond. Telehealth may provide benefits including high feasibility, acceptability and improved quality of life [74]. Lung function measured remotely compares well with lung function measured in hospital laboratories [75]. Further research is required to determine when and how to recommend telehealth [76].

## Discussion

For this work, we included members of the public including parents, caregivers and experts in CF. We identified unmet needs and priorities from a caregiver’s perspective. Revised hygiene safety and treatment recommendations for people with CF taking HEMT seem warranted. We assume that involving children and parents can improve communication, trust in research and treatment adherence; however, we could not include children and adults with CF yet. Involvement of children and adults with CF into PPI projects on a regular basis remains to be established at our center. We aim to extend our evolving PPI group to people with CF allowing their contribution in future CF research. Future work should consider (i) conceptual models for PPI and (ii) estimates of impact of PPI including costs or benefit of PPI [8, 77].

We acknowledge that this work has limitations in several aspects. In general, the interpretation of narrative reviews should be cautious as systematic grading the strength of recommendation based on evidence is usually not provided. For most of the suggestions made including those for hygiene prevention measures, evidence is scarce and largely based on expert views. Including input from multiple families and people at different stages of the disease would have added greater granularity. Lack of funding influenced communication in the PPI team and constrained regular exchange during working hours. Yet, written correspondence enabled working at times convenient for the team. A further limitation of PPI in CF is that face-to-face collaborative meetings are discouraged for people with CF or other muco-obstructive lung diseases due to potential risks of cross-infection [44].

We acknowledge the psychological and social burden of strict hygiene recommendations and the uncertainty in how HEMT might ultimately change these recommendations. There is an unmet need for updated evidence-informed hygiene guidance [78].

### Personal viewpoint of a family with two children with CF

For our children, the abovementioned statements, hygiene suggestions and behavior patterns have proved to be of value. The latter should be regarded as a possible implementation strategy, not as medical guidelines. Every family with children with CF is responsible for their approach to prevent infections.

## Conclusion

The use of HEMT improves and can stabilize lung disease but does not eliminate CF-related organ damage or treatment burden. Infection risk is reduced but persists, requiring ongoing hygiene and surveillance. Currently, daily hygiene and therapeutic procedures are time-consuming, yet remain the core of CF management despite HEMT. Digestion and thriving usually improve during HEMT, however, most people still need PERT and other supportive therapies. Long-term organ outcomes under HEMT remain uncertain.

The treatment and care of children and adults with CF remains extensive, complex and involves significantly more effort compared to their healthy peers. Continued, structured training of healthcare professionals, educators and families is needed to ensure sustained CF-specific, age-appropriate hygiene measures and medication. With or without modulator treatment adapted, PPI-informed guidelines for hygiene and childcare seem warranted.

Sustained and meaningful collaboration between patients, the public, and researchers can foster effective partnerships, ensuring that health research aligns with patient needs and healthcare for people with CF advances [79]. Public and patient involvement has proven valuable in identifying priorities and should be expanded to include children and adults. Public and patient involvement ensures bi-directional knowledge transfer between experts and those affected, allows further collaborative development of drugs, and intends to encourage the development of similar models beyond CF, especially for chronic congenital diseases.
